# Patient educational videos on T1 colorectal cancer

**DOI:** 10.1016/j.vgie.2023.08.009

**Published:** 2023-08-19

**Authors:** Nik Dekkers, Hao Dang, Jolein van der Kraan, James C.H. Hardwick, Alexandra M.J. Langers, Jurjen J. Boonstra

**Affiliations:** Department of Gastroenterology and Hepatology, Leiden University Medical Center, Leiden, The Netherlands

## Abstract

Video 1 Colorectal cancer: how does it develop and how can you detect it?

Colorectal cancer: how does it develop and how can you detect it?

Video 2 A polyp suspected to be colorectal cancer: what now?

A polyp suspected to be colorectal cancer: what now?

Video 3 Early-stage colon cancer with unfavorable features: what now?

Early-stage colon cancer with unfavorable features: what now?

## Educational Videos

This study provides a structured and informative overview of the journey of patients with T1 colorectal cancer (T1CRC) in 3 distinctive videos.

The first video (Video 1, available online at www.videogie.org) provides a general introduction to colorectal cancer. This video discusses how colorectal cancer develops and how early forms can be intercepted. It serves as a preamble for the following video on polyps with suspected T1CRC.

The second video (Video 2, available online at www.videogie.org) applies to all patients with a polyp suspected for T1CRC. This video discusses the following topics: the uncertainty of a T1CRC diagnosis prior to removal, the necessity of an en bloc complete removal using local resection techniques, and the 3 possible histological outcomes after local resection of a polyp that is suspected to be CRC. For patients with a histologically confirmed T1CRC (scenarios II & III in the video), we then discuss the considerations of whether to undergo additional treatment after local resection.

The third video (Video 3, available online at www.videogie.org) provides additional information that is only applicable to patients with a high-risk T1CRC (scenario III). This video discusses the following topics: benefits, disadvantages and nature of additional treatment, the influence of tumor location on surgical morbidity, refraining from additional treatment, and follow-up.

Adobe Illustrator and Adobe After Effects (both from Adobe Inc, San Jose, Calif, USA) were used to generate and refine the storyboard and to create the animation videos. All graphics, images, and videos are completely original. In the context of improving the quality of patient care, we have also asked a panel of 13 patients with (suspected) T1CRCs to share their opinion about these educational videos (see Table 1 for the results).

**Table 1:** Feedback of 14 patients

Question	Patients (n = 14)
**How understandable was the content of these videos?***Mean score out of 10*	9.4
**What did you think of the pace of these videos?**	
Too slow	0
Good	14 (100)
Too fast	0
**Did these videos improve the understanding of your disease?***Yes*	9 (64.3)
**Do you think these videos are a useful addition to the physician’s consultation?***Yes*	12 (85.7)
**Would you recommend a future patient to watch these videos?***Yes*	14 (100)
**What do you think is the preferred timing to show patients these videos?**	
Prior to consultation∗	5 (35.7)
During consultation	4 (28.6)
After consultation	5 (35.7)

Values are n (%) unless otherwise defined.

∗ All 14 reviewers indicated that a health care professional should introduce the possibility of a cancer diagnosis in person, prior to showing these videos.

## Discussion

The most complex decisional moment for patients with T1CRC is the decision to either proceed to additional treatment after local resection or to refrain from further treatment. Because of the complexity of this decision, a large proportion of these videos is devoted to this moment. Patients and their treating physician will have to weigh the benefits and disadvantages of additional treatment. The disadvantage of undergoing additional treatment is the risk of treatment-related morbidity and mortality, which depends on the type of additional treatment and the patient’s fitness. The benefit of additional treatment is reduction of the oncological risk, which includes reducing the risk of lymph node metastases (LNM) and disease recurrence. The current risk stratification model for LNM stratifies patients with T1CRC based on the absence or presence of 1 or more histological high-risk features.^1^ This model stratifies patients with T1CRC into low-risk (favorable features, scenario II) and high-risk (1 or more unfavorable features, scenario III) for LNM. T1CRCs with high-risk features also have an increased risk of disease recurrence after endoscopic resection, which is approximately 7% to 12.5%, whereas the risk of disease recurrence after endoscopic resection of a low-risk T1CRC is approximately 0.7% to 3.1%.^2^^,^^3^

If the possible disadvantages of additional treatment do not appear to outweigh the potential benefits, refraining from additional treatment can be the best option. This is generally the case for patients with a low-risk T1CRC (scenario II) because of their overall low benefit from additional treatment. In patients with high-risk T1CRC (scenario III), the decision to undergo additional treatment can be more challenging. Current guidelines recommend additional oncological resection after local resection of a high-risk T1CRC because of the higher oncological risk.^1^^,^^4^ Nevertheless, even for high-risk T1CRCs, the absolute risk for LNM or disease recurrence can still be considered relatively low.^5^ When using the above-mentioned risk stratification model, more than 80% of patients with a high-risk T1CRC referred for oncological resection turn out not to have LNM, despite being exposed to the risk of surgical morbidity and mortality.^5^ As a result, an increased tendency can be observed to refrain from additional treatment after local resection of T1CRC.^6^ Video 3 provides a summary of the aforementioned considerations, specifically for patients with a high-risk T1CRC for whom this decisional moment is most complex.

The absolute risk for LNM or disease recurrence varies greatly between patients, especially in the high-risk group. The recurrence risk of an individual patient depends on many different factors, including the number of high-risk features present and tumor location.^2^^,^^3^ Because we aimed to create videos that are applicable for all patients with T1CRC, we deliberately chose not to mention the absolute risk percentages.

## Conclusion

The educational videos in this study provide patient information about T1CRC and illustrate the different steps in the treatment of a polyp suspected to be colorectal cancer. They also address the considerations to opt for or refrain from additional treatment after local resection. Based on patient feedback, we believe that implementation of these videos might support the shared decision-making process, which is often complex in patients with T1CRC.

## Disclosure

This research was supported by the 10.13039/501100001717Leiden University Fund (reference number W20323-2-34). The funding party played no role in the design, writing, or decision to submit. All authors disclosed no financial relationships.

## Supplementary data

Video 1 Colorectal cancer: how does it develop and how can you detect it?

Video 2 A polyp suspected to be colorectal cancer: what now?

Video 3 Early-stage colon cancer with unfavorable features: what now?
